# Dr. William George Bacot

**Published:** 1910-09-24

**Authors:** 


Dr. William George Bacot, at Bournemouth. He took his M.R.C.S. and L.S.A. in 1851, and his L.R.C.P. in 1872. In 1867 he became a Fellow of the R.C.S., and, studying at the University of St. Andrews, graduated M.D. in 1873. He was a student at University College Hospital, and at one time held the post of house-surgeon at the Dorset County Hospital.

